# Composition and Interactions among Bacterial, Microeukaryotic, and T4-like Viral Assemblages in Lakes from Both Polar Zones

**DOI:** 10.3389/fmicb.2016.00337

**Published:** 2016-03-18

**Authors:** Aguirre de Cárcer Daniel, Carlos Pedrós-Alió, David A. Pearce, Antonio Alcamí

**Affiliations:** ^1^Centro de Biología Molecular Severo Ochoa, Consejo Superior de Investigaciones Científicas, Universidad Autónoma de MadridMadrid, Spain; ^2^CSIC, Institut de Ciències del MarBarcelona, Spain; ^3^British Antarctic Survey, Natural Environment Research CouncilCambridge, UK; ^4^Faculty of Health and Life Sciences, University of NorthumbriaNewcastle Upon Tyne, UK; ^5^University Center in SvalbardLonyearbyen, Norway

**Keywords:** bacteria, microeukaryotes, T4 phages, pyrosequencing, diversity, lakes, polar

## Abstract

In this study we assess global biogeography and correlation patterns among three components of microbial life: bacteria, microeukaryotes, and T4-like myoviruses. In addition to environmental and biogeographical considerations, we have focused our study on samples from high-latitude pristine lakes from both poles, since these simple island-like ecosystems represent ideal ecological models to probe the relationships among microbial components and with the environment. Bacterial assemblages were dominated by members of the same groups found to dominate freshwater ecosystems elsewhere, and microeukaryotic assemblages were dominated by photosynthetic microalgae. Despite inter-lake variations in community composition, the overall percentages of OTUs shared among sites was remarkable, indicating that many microeukaryotic, bacterial, and viral OTUs are globally-distributed. We observed an intriguing negative correlation between bacterial and microeukaryotic diversity values. Notably, our analyses show significant global correlations between bacterial and microeukaryotic community structures, and between the phylogenetic compositions of bacterial and T4-like virus assemblages. Overall, environmental filtering emerged as the main factor driving community structures.

## Introduction

The functioning of aquatic ecosystems is heavily dependent on the microbial food web (Azam et al., 1983), which consists of several components with different cellular (bacteria, archaea, and eukaryotes) or non-cellular (viruses) organizations. Polar freshwater environments embody some of the least human-impacted habitats on the planet (Convey and Stevens, 2007), and normally represent simple ecosystems with truncated food webs dominated by microorganisms. Arctic and Antarctic freshwater environments share common features, such as extreme annual cycles of temperature, sunlight, and ice phenology, and yet they are separated by long geographical distances. These characteristics make these environments a unique model to shed light on fundamental questions of aquatic microbial ecology, such as how communities vary across spatial scales and environmental gradients, or the association among different components of microbial life.

Previous studies assessing the links between different components of microbial life have focused on local patterns, such as the microbial inventory of a single coral reef ecosystem (McCliment et al., 2012), or the time-series analysis of a specific marine site (Jones et al., 2013; Chow et al., 2014). All these studies reported co-occurrence patterns among members of the different components of microbial life. Another significant question in aquatic microbial ecology is the importance of dispersal limitations (Martiny et al., 2011). However, most studies tackling this issue have focused on marine samples, presumably showing enhanced connectivity related to global oceanic circulation (Sul et al., 2011; Ghiglione et al., 2012). On the other hand, the question of whether microbial species in freshwater bodies from both poles are the same or different is also of interest from the viewpoint of biogeography and genetic exchange (Bano and Hollibaugh, 2002).

The aim of this study is two-fold; first, to assess if similar microbial communities populate freshwater bodies from both polar zones. Second, to test whether the previously observed co-occurrence patterns among components of microbial life appear at a global scale, or instead represent local phenomena. To do so, we have studied the resident microbial communities from four Arctic lakes located in the Svalbard archipelago, and nine Antarctic lakes sampled across a latitudinal transect along the Antarctic Peninsula. Using a massive parallel sequencing approach targeting phylogenetic marker genes, we have studied the community structures of three components of microbial life: bacteria, microeukaryotes, and viruses. Since the latter lack a common phylogenetic marker gene, we have focused on the T4-like myovirus group. This group represents a diverse and abundant (Filée et al., 2005; Williamson et al., 2008) group of bacteria-infecting viruses (Wichels et al., 1998; Clokie et al., 2010) amenable to the proposed approach based on their g23 major capsid gene, which has been shown to serve as a phylogenetic proxy (Comeau and Krisch, 2008). After obtaining the community profiles, we carried out several analyses, both at the OTU level and based on overall community phylogenetic compositions, to study the relationships among the three components of microbial life studied, and with respect to biogeographical and limnological (both physical and chemical) factors.

## Materials and methods

### Sampling and community DNA extraction

Planktonic samples were taken from freshwater bodies in the Arctic (Spitsbergen Island, Svalbard archipelago, Norway), and along the Antarctic Peninsula (Table 1, Supplementary Figure 1). Lake Nordammen (SvL1) was completely frozen (samples represent a combination of existing melted top ice from three different sites), while Lake Tenndammen (SvL2) is a shallow lake with frozen surface at the time of sampling. All the other lakes had open waters at the time of sampling and representative samples of the water column were taken at different depths, except for Limnopolar lake where water was taken from 4 m depth. All the lakes were sampled around summer. A portable probe was deployed *in situ* before sampling the Antarctic sites to measure several limnological variables (Temperature, Conductivity, pH, Chlorophyll; Supplementary Table 1). Lake Green's measurement was deemed unreliable due to technical issues and hence data from this lake were removed from those analyses including limnological data.

**Table 1:** **Location, sampling date, and characteristics of the sampled environments**.

	**Pole**	**Position^a^**	**Date**	**Zoogenic input^b^**
Lake Domo^*^	Antarctic	−62.64 −60.97	03/02/2010	Very low
Lake Refugio^*^	Antarctic	−62.65 −61.00	03/02/2010	Next to sea elephant colony
Lake Limnopolar^*^	Antarctic	−62.66 −61.10	01/02/2010	Very low
Caleta Cierva	Antarctic	−64.16 −61.01	21/01/2010	Nearby seabird colonies
Green Island	Antarctic	−65.31 −64.15	23/01/2010	Accessible to seabirds
Biscoe Point	Antarctic	−65.43 −65.48	29/01/2011	Very low
Pourquoi-Pas Island	Antarctic	−67.66 −67.25	27/01/2011	Very low
Avian Island	Antarctic	−67.76 −68.88	25/01/2010	Nearby seabird colonies
Horseshoe Island	Antarctic	−67.84 −67.19	25/01/2011	Very low
IR2	Arctic	78.04 13.69	27/09/2011	Frequent seabird activity
Lake Tunsijøen(IR1)	Arctic	78.05 13.65	26/09/2011	Frequent seabird activity
Lake Nordammen(Svl1)	Arctic	78.63 16.63	05/06/2012	Accessible to seabirds
Lake Tenndamen(Svl2)	Arctic	78.10 15.03	06/06/2012	Accessible to seabirds

a *Lat Long*.

b *in situ observations*.

* *Lakes in Byers Peninsula, Livingston Island*.

Single ninety liter samples from each water body were filtered through a 30 μm nylon mesh. Subsequent filtration by 0.45 μm tangential flow filtration (TFF) using a Centramate holder (Pall) separated the free viral community (defined as the < 0.45 μm fraction) from the cellular (and associated virus) fraction (defined as the fraction between 0.45 and 30 μm). Viral fractions were subsequently concentrated 100 times by 70-kDa TFF as described (López-Bueno et al., 2009; Aguirre de Carcer et al., 2015). All samples were preserved at −20°C/−80°C prior to DNA extraction. Cellular fraction community DNA was extracted using PowerSoil DNA isolation kit (MoBio, Carlsbad, CA) according to the manufacturer's instructions. Viral DNA was obtained from frozen stocks thawed at 4°C, and passed through a 25% sucrose cushion by centrifugation for 16 h at 60,000 g and 4°C. The pellets were re-suspended in 10 mM Tris pH 8, 1 mM EDTA, and filtered using a 0.45 μm syringe filter. Viral concentrates were then treated with DNAse I (500 U.ml^−1^), Nuclease S7 (420 U.ml^−1^), RNAse A (100 μg.ml^−1^) and RNAse H (2 U per reaction) for 30 min at room temperature to remove free nucleic acids. Nuclease reactions were stopped with 12 mM EDTA/2 mM EGTA, and viral capsids and envelopes were then disrupted with SDS (0.5%) and proteinase K (200 μg.ml^−1^) treatment. Finally, viral DNA was extracted with phenol-chloroform and ethanol precipitated.

### PCR amplification of community marker genes, and massive parallel sequencing

Bacterial 16S rRNA marker genes were amplified from the cellular fractions using primers 8F15B (5′-[RocheAdapterB]AGAGTTTGATCCTGG-3′) and 515R14AM (5′-[RocheAdapterA]-bc-TTACCGCGGCTGCT-3′; (Aguirre de Cárcer et al., 2011b)). All PCR reactions were carried out using 1 μl of template DNA, 0.5 μl Phusion High-fidelity polymerase (NEB), 20 nmol dNTPs, 20 pmol of each primer, 1.5 μl DMSO, 0.4 mM MgCl_2,_ in a final volume of 50 μl. Reaction conditions included an initial denaturation step of 30 s at 98°C, followed by 25 cycles of 10 s at 98°C, 30 s at 53°C, 30 s at 72°C, and a final elongation step of 5 min at 72°C. For the analysis of the microeukaryotic and T4-like viral assemblages we followed a validated two-step barcoding strategy (Aguirre de Cárcer et al., 2011b) that allows a universal set of bar-coded sequencing primers to be appended to an amplified PCR product without introducing discernible biases. In the first step, one of the target-specific primers is modified to include a linker sequence. After amplification, a second primer consisting of the bar code and linker is used to tag the amplicon. The eukaryotic community in the cellular fractions was analyzed using primer sequences targeting the 18S rRNA gene (Bates et al., 2013; F515; 5′-GTGCCAGCMGCCGCGGTAA-3′ and R1119 5′-GGTGCCCTTCCGTCA-3′). The T4-like virus communities in both cellular and free viral fractions were assessed using primer sequences (Filée et al., 2005) targeting the g23 major capsid protein gene (MZIA1bis 5′-GATATTTGIGGIGTTCAGCCIATGA-3′ and MZIA6 5′-CGCGGTTGATTTCCAGCATGATTTC-3′). In this case, we performed an initial amplification using the unmodified (no linker or 454-adaptor sequences) primers (45 cycles, melting temperature of 50°C), followed by agarose gel extraction of DNA bands of appropriate size. The resulting products were re-amplified using the modified primers (10 cycles), at this point linking with the two-step protocol. The primer pairs employed in this study have been reported to target most known sequences within their target groups. Final concentrations of PCR products were measured using a PicoGreen dsDNA Assay Kit (Life Tech.), equal amounts for each sample pooled, agarose gel-extracted using the QIAquick Gel Extraction Kit (QIAGEN), and sequenced using a Roche 454 FLX sequencer with titanium chemistry. Bacterial and Eukaryotic profiles from each lake were generated in triplicate (hence three different barcodes/reactions per sample) to mitigate potential reaction-level PCR biases (Bates et al., 2013). All sequences have been deposited at ENA under id PRJEB10639.

### Sequence processing and data analysis

The sequences obtained were denoised with ACACIA (Bragg et al., 2012) then processed using QIIME (Caporaso et al., 2010). Sequences were first assigned to each sample using their respective barcodes. They were next filtered for correct length and quality values (Maximum number of ambiguous bases; 5. Mean quality score; >25. Maximum homopolymer length; 6. Maximum mismatches in primer; 0. Chimera removal with *usearch*). Later, all sequences were grouped into operational taxonomic units (OTUs) at 0.97 distance thresholds using the Uclust algorithm, and OTUs not appearing in at least two replicates across each dataset were discarded to eliminate noise and possible artifacts. At this point, the results from the per-sample technical triplicates were pooled to obtain per-sample community profiles. The resulting sample-by-OTUs matrices were subsampled to the minimum number of sequences at any given site (independently for each marker gene dataset) to normalize sampling efforts, and singletons were removed (Aguirre de Cárcer et al., 2011a). Additionally, representative sequences from each bacterial and eukaryotic OTU (the most abundant sequence of that OTU across the dataset) were confronted against the Greengenes (DeSantis et al., 2006)/Silva (Quast et al., 2013) reference alignments (for 16S and 18S sequences respectively) for taxonomic affiliation. Shannon diversity indices, Chao1 richness estimates, and percentage of shared OTUs between samples were obtained using dedicated QIIME scripts.

Statistical and analytical procedures were carried out in *R* (R Core Team, 2015).

Overall differences between poles or sampling fractions (only for T4-like viruses) in terms of richness, diversity, percentage of shared OTUs, and relative abundance of major eukaryotic taxa were assessed using *t*-tests (t.test function).

The relationships between diversity values and latitude, limnological variables, or the relative abundance of major taxonomic groups were explored through linear regression. Bootstrapping was conducted to test whether or not the slope of the regression was significantly different from zero (*boot* function within package of the same name, Davison and Hinkley, 1997), and alternatively, performing a Wald test for multiple coefficients *(f.robftest* function from package *sfsmisc*).

The existence of correlation between percentages of shared OTUs and geographical distance or limnological profiles was assessed through Mantel tests (*mantel.randtest*, package *ade4*).

The initial exploration of community structure data was carried-out through double principal coordinates analysis (DPCoA) using functions from the *ade4* package. This is an ordination method that takes into account phylogenetic (genetic distance) relatedness between OTUs when explaining variation in the data, hence quantifying community dissimilarity based on phylogenetic relatedness. Statistical significance of *a priori* community groupings (e.g., pole of origin) was tested by between class analysis (BCA) and constrained double principal coordinates analysis (cDPCoA) (phylogenetic-aware) (Dray et al., 2015) available in *ade4*. Identification of limnological variables correlated with community structure was undertaken by analysis with instrumental variables (IV) (Baty et al., 2006) using the *pcaiv* function of the same package. Finally, associations among community structures derived from different marker genes, as well as with existing environmental data (limnological profiles, geographic distance, latitude) were assessed with Mantel tests using Bray-Curtis and Rao (phylogenetic-aware) distance matrices derived from the community profiles.

## Results

### OTUs richness and diversity

Samples from four water bodies in Spitsbergen Island (78°N, Svalbard, Norway) and nine water bodies along the Antarctic Peninsula (62°–67°S) were collected in three different years (Table 1, Supplementary Figure 1). From these samples, community DNA was prepared using both cellular and free virus fractions, and bacterial 16S rRNA, eukaryotic 18S rRNA and T4-like virus g23 major capsid protein genes were PCR-amplified and sequenced using Roche 454 technology. The strategy employed produced 78000 (16S), 23000 (18S), and 38000 (g23) sequences passing the initial quality control. Subsequent filtering resulted in 5302 ± 1040 (16S), 1805 ± 440 (18S), and 1514 ± 496 (g23) sequences per sample (Supplementary Table 1). However, we were unable to produce reliable g23 sequence data for a small subset of the sites and fractions (4 out of 26), and hence these data points were removed from some analyses. Clustering of sequences at 97% similarity produced 1548 (16S), 704 (18S), and 176 (g23) OTUs in total. The number of bacterial OTUs per sample ranged between 126 in Horseshoe Lake and 864 in Lake SvL1 (Supplementary Table 1). The number of singletons was moderate in all cases, resulting in Chao 1 estimates that were only about twice as large as the retrieved OTUs (ranging between 121 and 1466). Corresponding values were lower for eukaryotes with values ranging between 87 OTUs in Lakes Biscoe and SvL2 and 418 in Lake IR2 (average 197), and even lower for viruses. Interestingly, viral richness was higher in the cell-associated (average 33 OTUs) than in the free viral fractions (average 21 OTUs, Supplementary Table 1) although difference did not reach statistical significance (*p* > 0.05).

The microbial communities studied shared a noticeable percentage of OTUs: averaging 14, 10.2 and 5.8% for bacteria, eukarya and viruses respectively (Table 2). Communities from the same polar zone shared a significantly higher percentage of OTUs. The only exception was the Arctic bacterial assemblages, which only shared approximately 10% of their OTUs among themselves. In the case of T4-like viral assemblages, a higher percentage of shared OTUs between the free viral and cellular fractions arising from the same sample was observed when compared to the inter-lake average (31.4% vs. overall 5.8%, *p* < 0.0005, respectively). For the Antarctic bacterial and microeukaryotic data sets, we found no significant correlation between the percentage of shared OTUs and either geographical distance or limnological profiles based on temperature, conductivity, pH, and Chlorophyll (Supplementary Table 2).

**Table 2:** **Shared OTUs**.

	**16S**	**18S**	**g23**
Overall	14.0±4	10.2±2.4	5.8±11.6
Intra-Antarctic	19.4±4^***^	13.7±2.2^***^	5.7±2.2^*^
Intra-Arctic	10.1±1.1	11.7±2.8^*^	10.9±9.3^*^

*Numbers represent averaged values (%) of geometric means of the pairwise comparisons*.

* p < 0.05;

*** *p < 0.0005*.

*Paired t-tests. Comparison with respect to the overall shared OTUs*.

**Figure 1:**
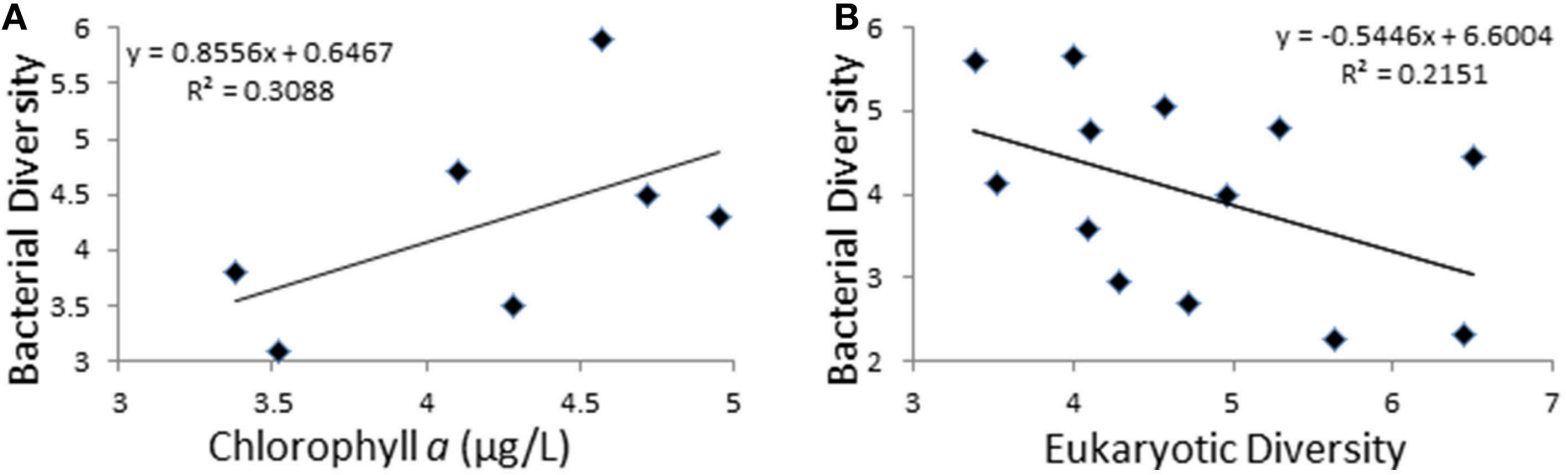
**Exploration of diversity trends. (A)** Relationship between bacterial diversity (Y-axis) and chlorophyll *a* concentration (X-axis). **(B)** Relationship between bacterial (Y-axis) and microeukaryotic diversity (X-axis). Diversity is represented by Shannon's indexes and Chlorophyll *a* is in μg/L.

Shannon's entropy index was used to study community diversity (Table 3). No significant differences were found when comparing diversity values between the two polar zones, either for the bacterial (Antarctic; 4.7 ± 1.1, Arctic; 4.7 ± 0.8) or microeukaryotic assemblages (Antarctic; 3.9 ± 1.1, Arctic; 4.0 ± 1.4). Also, for the Antarctic datasets, no correlation was observed between community diversity values and latitude along the studied transect. Moreover, observed diversity values and recorded limnological variables (Supplementary Table 2) only correlated in the case of 16S-based bacterial community diversity and Chlorophyll *a* content, where a positive correlation was found (Slope 0.85, *R*^2^ = 0.3), although the observed correlation only reached significance (*p* < 0.05) with one of the two statistical tests applied (Figure 1A). Strikingly, a negative correlation (Slope −0.54, *R*^2^ = 0.21) was observed between bacterial and microeukaryotic diversity values (Figure 1B), with the negative association reaching a noticeable degree of significance with both statistical tests applied (linear regression bootstrapping *p* < 0.05; Wald test *p* = 0.078). Subsequent analyses failed to show significant correlations between diversity indices of bacterial and microeukaryotic assemblages and the relative abundances of any major taxonomic groups.

**Table 3:** **Shannon's diversity indices**.

	**Bacteria**	**Eukarya**	**Virus^f^**	**Virus^c^**
Domo	4.28	2.95	NA	2.25
Refugio	6.50	4.46	NA	NA
Limnopolar	4.95	3.99	3.01	2.84
Cierva	3.37	5.61	NA	1.52
Green	6.45	2.31	2.63	2.39
Biscoe	4.72	2.68	0	2.16
Pourquoi-pas	4.10	4.76	0.39	1.94
Avian	4.57	5.04	2.28	2.29
Horseshoe	3.51	4.12	1.36	1.84
IR2	4.00	5.67	1.95	1.34
Tunsijøen IR1	5.29	4.80	1.32	1.45
Tenndamen SvL2	5.62	2.26	1.83	1.99
Nordammen SvL1	4.08	3.58	1.84	4.17

f *Free virus fraction*.

c *Cellular fraction*.

In the case of the T4-like viral assemblages (Table 3) no significant differences in diversity estimates were observed with regards to pole of origin. 16S and 18S-based values obtained for the same samples gave no significant correlations when compared to T4-like virus values. Moreover, geographical (distance, latitude) and limnological parameters available for the Antarctic sites did not show an influence on T4-like virus community diversity. Lastly, the cellular fractions were more diverse than their free viral fraction counterparts, yet such differences did not reach statistical significance (*p* = 0.11). One peculiar case was that of the free viral fraction of Lake Biscoe that consisted of a single OTU. This OTU was also detected at 11% relative abundance in the cellular fraction of Lake SvL1 in the Arctic suggesting that it was not an artifact.

### Taxonomic compositions

**Figure 2:**
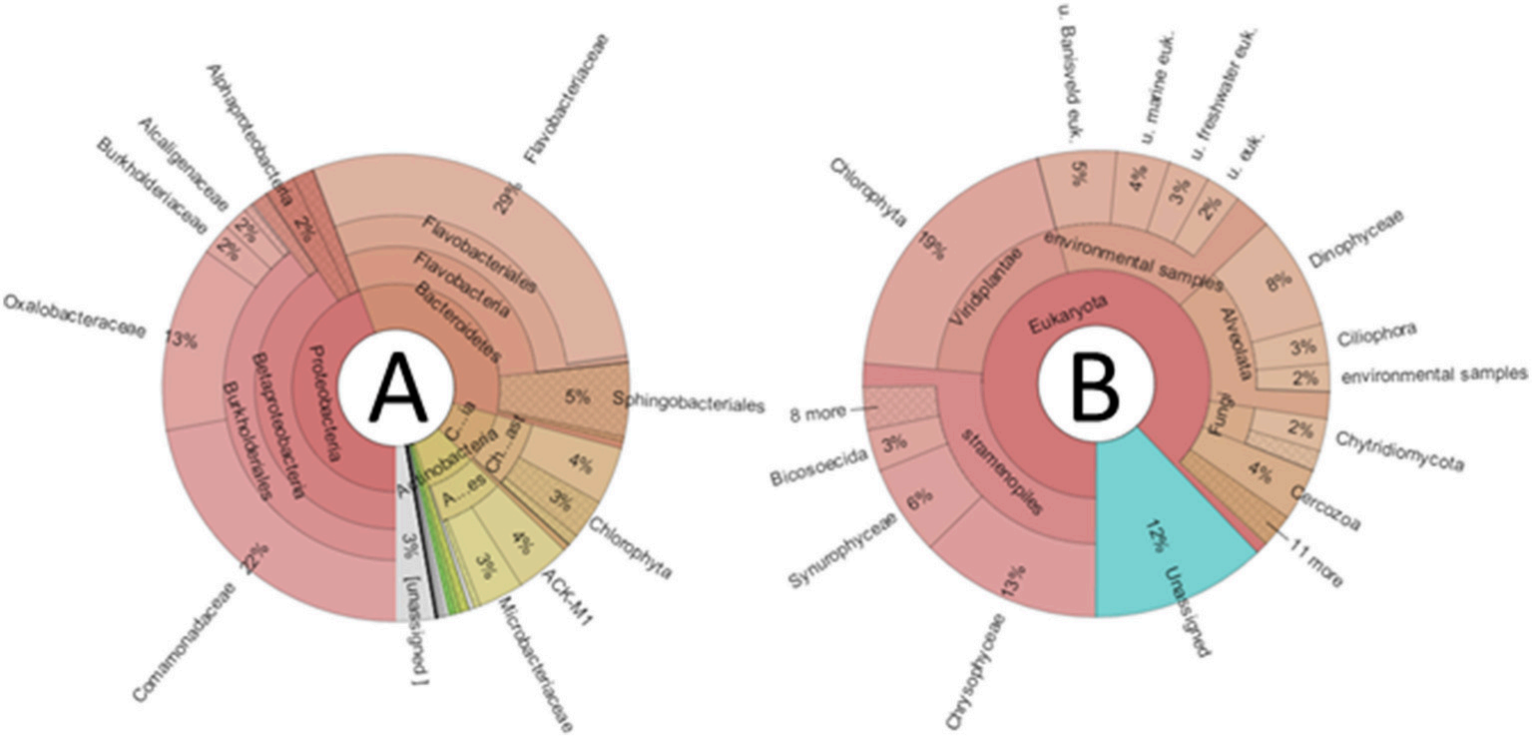
**Krona graphs depicting averaged relative abundances of taxonomic groups in the studied communities**. **(A)**, Bacteria; **(B)**, Eukarya; u, uncultured; euk, eukaryote.

**Figure 3:**
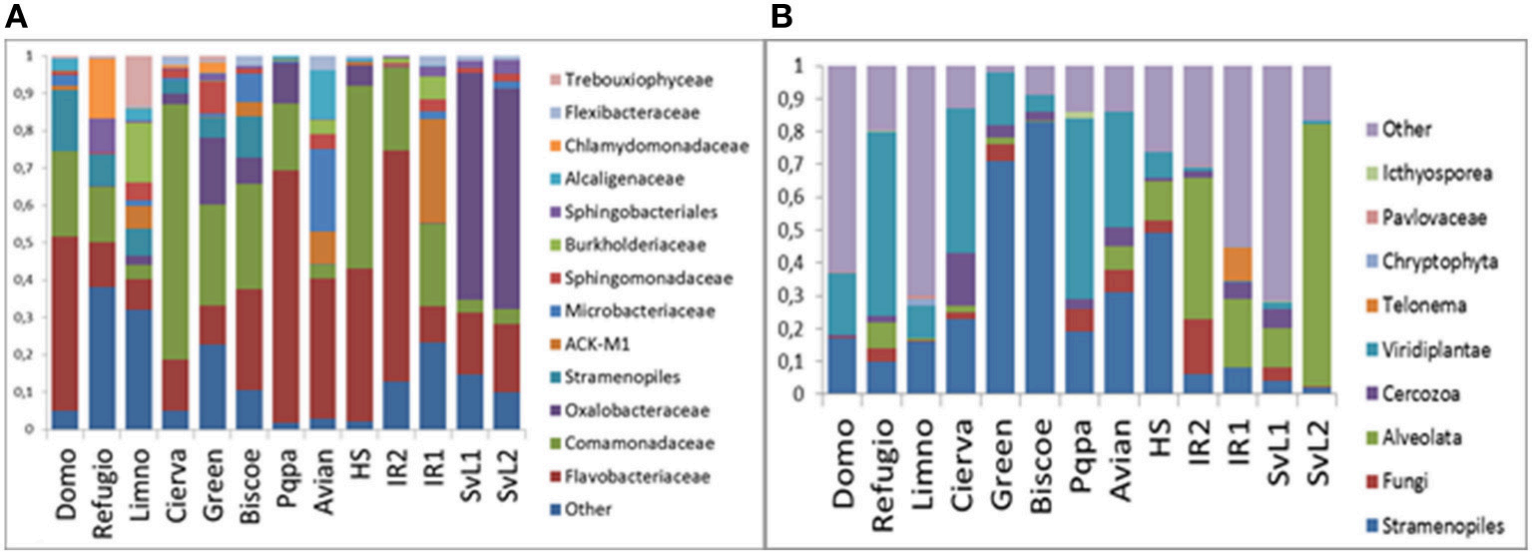
**Per-lake relative abundances of taxonomic groups in the studies communities**. **(A)** Bacteria. **(B)** Eukarya.

Bacterial community profiles (Figure 2A) were dominated by sequences classified as order Burkholderiales (39%), containing mainly representatives of families Oxalobacteriaceae (13%) and Comamonadaceae (22%). Bacteroidetes-related sequences were also very abundant (35%), dominated by Flavobacteriaceae (29%), and Sphingobacteriales (5%). Actinobacteria-related sequences were abundant (8%), mainly corresponding to the Microbacteriaceae (3%), and ACK-M1 (4%) clades. Finally, Cyanobacteria-related sequences averaged 8%, yet a closer inspection revealed that, although a few sequences belonged to the Synechococcophycidae and Nostocaceae clades, the great majority (94%) corresponded to algal chloroplast-related sequences. When the taxonomic composition was examined separately for each lake, substantial differences emerged (Figure 3A). The predominance of Betaproteobacteria and Bacteroidetes was apparent, but some lakes were dominated by the former, such as Cierva, Horsheshoe, SvL2, and Svl1, while others were dominated by Bacteroidetes, like Domo, Pourquois-Pas, and IR2. Chloroplasts appeared only in low-latitude Antarctic lakes. Finally, Actinobacteria were abundant in several Antarctic lakes but only in one Arctic lake. Interestingly, within the Betaproteobacteria, family Oxaloacetaceae was the most abundant in two Arctic lakes (SvL1 and Svl2), while Comamonadacea was the most abundant family in most other lakes.

Globally, microeukaryotic assemblages (Figure 2B) were dominated by sequences classified as Stramenopiles (26%), Viridiplantae (19%), and Alveolata (15%). A major difference between the two polar zones became evident when the composition was examined for the different lakes separately (Figure 3B): Arctic lakes were clearly dominated by Alveolata while the Antarctic lakes had mostly Viridiplantae and Stramenopiles, with differences reaching statistical significance (*p* < 0.05). The Stramenopiles belonged mostly to the Chrysophyceae (12%) and Ochromonadaceae (6%). The Viridiplantae were mostly Chlorophyta, and the Alveolata mostly Dinophyceae (8%). Some Antarctic lakes were dominated by Viridiplantae while others were dominated by Stramenopiles. Other groups were present in some lakes only, and in smaller proportions. Cercozoa were one such group. They were abundant in two Arctic and one Antarctic lake and present in a few more lakes. There were also sequences related to fungi. In particular, one extremely abundant (52.4%) OTU in Lake SvL2 related to the Basidiomycota, and Chytridiomycota-affiliated sequences in lake IR2 (12.5%). Another interesting case was that of a *Telonema* OTU that was very abundant in Lake IR1. *Telonema* is a widely distributed marine heterotrophic flagellate that forms a deep branch in the tree of eukaryal life. Finally, an average 13% of eukaryotic sequences could not be properly assigned, and 17% were related to uncultured eukaryotes.

Overall, bacterial community compositions for the studied environments were dominated by members of the Burkholderiales, Bacteroidetes, and Actinobacteria, the same groups found to dominate both Polar and non-Polar freshwater ecosystems (Newton et al., 2011; Logares et al., 2013; Barberán and Casamayor, 2014; Vick-Majors et al., 2014). The same was true for the microeukaryotic assemblages, where the dominance of Stramenopiles, Chlorophyta, and Alveolata, is in line with previous knowledge that most eukaryotes in other Antarctic lakes were photosynthetic microalgae (Wilkins et al., 2013; Vick-Majors et al., 2014). Phototrophic microeukaryotes accounted for about 60% of the eukaryotic sequences. On the other hand, 16S sequences provided very few sequences of free-living cyanobacteria and a substantial amount of chloroplast sequences. This indicates that the primary producers in the studied pelagic ecosystems were eukaryotes, which is in contrast to the dominant role of Cyanobacteria in the benthos of Antarctic freshwater systems (Wharton et al., 1983; Vincent, 2000). However, our results are in agreement with the fact that viral metagenomes from polar freshwater environments were found to be dominated by likely microeukaryote-infecting viruses (López-Bueno et al., 2009; Aguirre de Carcer et al., 2015), and with results from microscopy observations (Izaguirre et al., 1998).

### Community structure

**Figure 4:**
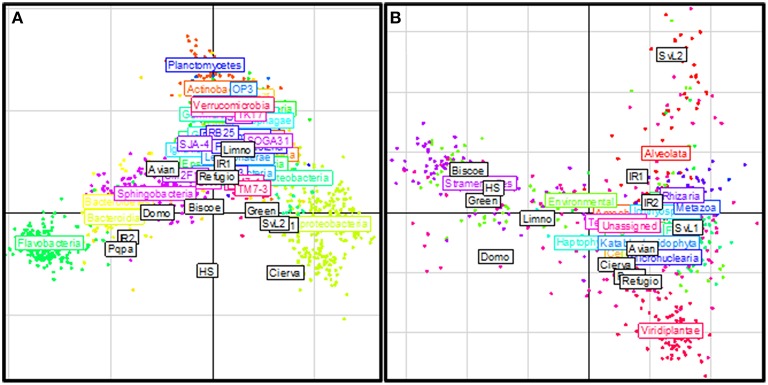
**Phylogenetic composition similarities between polar freshwater assemblages**. The figures depict results from Double Principal Coordinates Analyses (DPCoA) based on 16S **(A)** and 18S **(B)** marker gene profiles. The analysis ordinates the sites (white boxes) based on the phylogenetic distances and per-site relative abundances of OTUs. The position of each point (OTU) represents its association with each site. Points are colored and collectively labeled based on their taxonomic affiliation.

Double principal coordinates analyses were used to explore the relationships between phylogenetic community compositions at each site. The major source of variation in the bacterial data set (Figure 4A) related to the relative proportions of sequences affiliated as Flavobacteria and Betaproteobacteria. Lakes Avian, Domo, IR2, and Pourquois-Pas showed increased abundances of Flavobacteria and reduced abundances of Betaproteobacteria, whereas Cierva, SvL1, SvL2, and Green exhibited the opposite pattern. On the other hand, variation in the microeukaryotic data set was principally related to the relative proportions of sequences affiliated to the Stramenopiles, Alveolata, and Viridiplantae clades (Figure 4B). As already mentioned Antarctic communities had significantly (*p* < 0.05) lower relative abundances of Alveolata-affiliated sequences and higher proportions of those affiliated to the Stramenopiles and Viridiplantae clades, when compared to the studied Arctic sites. Furthermore, the results confirmed two opposing clusters of Antarctic sites along a Stramenopiles-Viridiplantae axis.

For the remaining exploration of the datasets, we carried-out two types of analyses (Table 4): those based solely on OTUs relative abundances (Mantel tests of Bray-Curtis distances, Between Class Analysis), and analyses based on both OTUs relative abundances and phylogenetic relatedness between OTUs (Mantel tests of Rao distances, constrained Double Principal Coordinates Analysis). First, we examined the relationship between the composition of the cellular components and both geographical distance and limnological parameters (Table 4A). We found no significant association between either component and geographic distance or latitude. On the other hand, we observed a significant correlation between bacterial community structure and the limnological parameters recorded in the Antarctic sites, and marginal association (*p* = 0.068) between the same parameters and the microeukaryotic assemblages. In both cases, associations seemed to be related to temperature and conductivity (analysis with instrumental variables based on Bray-Curtis distances; *p* < 0.05). Nevertheless, comparisons became statistically not significant when taking into account the overall phylogenetic composition of the communities.

**Table 4:** **Community structure analyses**.

**Comparison**	**Test**	**Sign**	**Comparison**	**Test**	**Sign**
**A. Antarctic vs. Environment:**			**B1. Correlation between assemblages:**		
Bacteria vs. Geo. Distance	Mantel(BC)	na	Bacteria Vs. Microeukaryotes	Mantel(BC)	^***^
	Mantel(Rao)	na		Mantel(Rao)	na
Bacteria vs. Latitude	Mantel(BC)	na	Bacteria Vs. Virus^F^	Mantel(BC)	na
	Mantel(Rao)	na		Mantel(Rao)	na
Bacteria vs. Physicochem.	Mantel(BC)	^**^	Bacteria Vs. Virus^C^	Mantel(BC)	na
	Mantel(Rao)	na		Mantel(Rao)	^**^
Microeukaryotes vs. Geo.Distance	Mantel(BC)	na	Microeukaryotes Vs. Virus^F^	Mantel(BC)	na
	Mantel(Rao)	na		Mantel(Rao)	na
Microeukaryotes vs. Latitude	Mantel(BC)	na	Microeukaryotes Vs. Virus^C^	Mantel(BC)	na
	Mantel(Rao)	na		Mantel(Rao)	na
Microeukaryotes vs. Physicochem.	Mantel(BC)	^*^			
	Mantel(Rao)	na			
			**C. Between-poles:**		
**B2. Virus (other):**					
			Bacteria	BCA	^**^
				cDPCoA	na
Between lakes	BCA	^**^	Microeukaryotes	BCA	^**^
	cDPCoA	^**^		cDPCoA	^**^
Virus^F^ vs. Virus^C^	BCA	^**^	Virus	BCA	^**^
	cDPCoA	na		cDPCoA	^**^

*Tests: Mantel(BC), [Mantel test based on Bray-Curtis distances]; Mantel(Rao), [Mantel test based on Rao distances{Phylogenetic}]; BCA, [Between class analysis]; cDPCoA, [Constrained Double Principal Coordinates Analysis {Phylogenetic}. Sign: na (p > 0.1)*,

*** *(p < 0.001)*,

** *(p < 0.05)*,

* *(p < 0.1)*.

F *Free virus fraction*,

C *Cellular fraction*.

Next, we analyzed the correlations among the different microbial components (Table 4B). Bacteria and microeukaryotic assemblages showed a significant correlation employing Bray-Curtis distances, yet statistical significance disappeared when using Rao distances (phylogenetic-aware). On the contrary, bacterial and viral cellular fractions showed a significant correlation with Rao distances but not with Bray-Curtis distances. As expected, cellular and free virus fraction T4-like virus assemblages from the same lake were more similar to each other than to those of the other lakes, also when accounting for the genetic similarities among sequences. Interestingly, we observed a significant discrimination between virus assemblages arising from either cellular or free viral fractions with Bray-Curtis distances, although such differences disappeared when Rao distances were considered.

Finally, we examined potential differences between the two polar zones (Table 4C). Bacterial assemblages from the same pole were more similar to each other than to those of the other pole, yet the overall genetic diversity of both poles was undistinguishable. In the cases of the microeukaryotic and T4-like virus assemblages, on the other hand, both poles were shown to harbor distinct community structures, also when accounting for the genetic diversity of the OTUs.

## Discussion

### Composition of polar freshwater microbial communities

The phylogenetic analysis clustered the lakes on the basis of their microeukaryotic composition, with Arctic lakes bearing higher proportions of Alveolata/Dinophyceae, and Antarctic lakes partitioned along a Viridiplantae (Avian, Cierva, Pourquois-Pas, and Refugio) vs. Stramenopiles (Limnopolar, Biscoe, Horseshoe, Green, and Domo) axis. This second axis roughly matches the classification of the lakes according to their higher or lower Chlorophyll concentration (Supplementary Table 2). Interestingly, this partitioning is also in line with that reported by Izaguirre et al. (1998) for lakes in Hope Bay (Antarctica), where trophic status was shown to impact microeukaryotic assemblages: more oligotrophic lakes were dominated by Chrysophyceae (Stramenopiles), and more eutrophic lakes were dominated by Chlorophyceae (Viridiplantae). These authors reported that trophic status of the freshwater bodies in Hope Bay depended strongly on their proximity to bird colonies. In the present study most lakes did not appear to have a strong influence of marine birds and mammals. However, partitioning along the abovementioned axis correlated with estimated zoogenic input (Table 1). Moreover, Lake Refugio was surrounded by substantial numbers of elephant seals, likely explaining the very high value of Chlorophyll in this lake and the dominance of Viridiplantae.

We detected significant differences between T4-like virus assemblages arising from the cellular and free virus fractions. However, such differences appeared only at the level of OTUs, and not when accounting for the overall phylogenetic compositions of the fractions. These observations may indicate that different phylogenetically-related viral OTUs have a preference to localize in one or the other fraction. This fact may have important methodological consequences, since most protocols used to study overall viral assemblages (e.g., through viral shot-gun metagenomics) rely on the study of the free viral fraction exclusively, in order to reduce the massive proportion of undesired bacterial DNA in the resulting datasets (López-Bueno et al., 2009; Fancello et al., 2013; Brum and Sullivan, 2015). Also, the free virus fraction was less diverse than the cellular fraction (e.g., intra-cellular and membrane-attached viruses). Moreover, a significant correlation was found between bacterial and T4-like viral assemblages present in the cellular fractions. Altogether, these results indicate that studying solely free viral fractions may result in a rather biased picture of the ecology of viruses.

### Limnological parameters drive antarctic community structures

We could not find evidence for latitude or geographical distance having a significant influence on the community composition of Antarctic sites (Table 4A). This was true for both the bacterial and the microeukariotic components. On the other hand, limnological parameters correlated with both bacterial and microeukaryotic community structure (Table 4A). The same weak or inexistent short to mid-range biogeographical patterns in microbial communities, combined with strong environmental filtering have previously been observed in similar ecosystems, such as Antarctic lakes (Logares et al., 2013), or sub-arctic and Arctic marine environments (Winter et al., 2013). Subsequent analyses revealed that both conductivity and temperature co-varied with community structure. However, it is important to note that in the present dataset temperature and conductivity were correlated (Supplementary Table 1). The influence of temperature was not unexpected, since it had previously been shown to affect bacterial community structure in northern European lakes (Lindström et al., 2005). The effects of temperature and conductivity were significant exclusively on the Bray-Curtis distances, but they did not affect overall phylogenetic compositions (Table 4A). The fact that the latter remained unaffected by environmental variables likely indicates the existence of ecologically-redundant, phylogenetically-related, OTUs fit to slightly different limnological scenarios. We had anticipated significant pH effects, as they had previously been reported in studies of high altitude (Barberán and Casamayor, 2014; pH range 4.5–9), northern Europe (Lindström et al., 2005; pH 5.5–8.7), and Wisconsin lakes (Yannarell and Triplett, 2005; pH 5.4–8.6). It is possible that we did not detect pH-related effects due to the reduced dataset size. On the other hand, pH range along our sites did not reach two units (5.8–7.7), while in all abovementioned studies the range exceeded 3 units.

### Bipolar patterns in community structures

The overall percentages of shared OTUs were considerable (between 6 and 19%), further substantiating the idea that while each lake has a particular community structure, many lakes can harbor the same OTUs (Newton et al., 2011). Nevertheless, the percentages of shared bacterial OTUs between lakes from different poles were noticeably lower than those observed between marine Arctic and Antarctic bacterial assemblages (*ca*. 20–30%) (Ghiglione et al., 2012), suggesting a stronger effect of water-borne bacterial dispersal and (or) a greater variability in the physicochemical environment between lakes vs. ocean samples. In particular, Ghiglione et al. (2012) found indications that deep water circulation might be responsible for dispersal of OTUs from one polar ocean to the other. Lakes are not communicated by oceanic circulation and, thus, dispersal can only occur through the air or through migrating Arctic terns. In this regard, it is not surprising that the number of shared OTUs is lower in freshwater bodies than in marine polar areas. Additional examples of similar or even identical sequences retrieved from both polar marine areas exist for example for Nitrosospira {Hollibaugh et al., 2002 #375} or for ammonia-oxydizing Chrenarchaeota {Kalanetra et al., 2009 #377}. In fact one of the latter sequences was found to be dominant during the dark winter period in the Arctic Ocean {Alonso-Saez et al., 2012 #378}. Another interesting example is that of cyanobacteria isolated from mats in ponds from both Polar zones, Nadeau et al. found that psychrophilic strains had almost identical sequences in their 16S rDNA, while psychrotolerant strains were more different.

There were no differences in diversity estimates between poles for the three components of microbial life. Nevertheless, the results based on community structure, phylogenetic composition, and shared OTUs, all point to a consistent biogeographical pattern segregating both poles. The only exception was that the bacterial assemblages were not significantly different in terms of phylogenetic content. This pattern had already been observed for the bacterial assemblages of Antarctic vs. Scandinavian lakes (Logares et al., 2013), as well as for the bacterial and microeukaryotic assemblages present in cryoconite holes from both poles (Cameron et al., 2012).

### Relationships among the three microbial components

We found a significant correlation between bacterial and microeukaryotic community structures. However, such link disappeared when accounting for the overall phylogenetic content of the communities. These results indicate that while the overall phylogenetic compositions of each of these microbial groups are not coordinated, there are particular associations between several of their OTUs. Similar findings have recently been reported for a single marine site (Chow et al., 2014). While these signals may simply reflect the associated OTUs' preferences for similar environmental parameters, they could also indicate true biotic interactions.

Our analyses also showed a significant correlation among bacterial and T4-like virus assemblages present in the cellular fractions, although in this case only when accounting for the phylogenetic relatedness among OTUs. Links between viral and bacterial community compositions had previously been detected in a large scale study of sub-polar and Arctic marine sites (Winter et al., 2013). The results from the latter study, however, were not conclusive, since observed links were regionally restricted and failed to show such pattern for the entire dataset. The circumstance that we did not observe such pattern using the free virus dataset may relate to increased noise, since this community may be dominated by viral strains recently arising from exponential outbursts. Nevertheless, the fact that such pattern was detected when accounting for phylogenetic relatedness among OTUs is in line with the idea that many viral strains may infect more likely phylogenetically-related hosts, although this predominant idea has not yet been properly addressed (Flores et al., 2011).

## Conclusion

This study represents, to our knowledge, the first massive parallel sequencing-based assessment of three components of microbial life; bacteria, microeukaryotes, and T4-like viruses. In this regard, we believe this study offers an important complement to recent efforts to monitor these relationships following time-series analysis of a single marine ecosystem employing Automated Ribosomal Intergenic Spacer Analysis (ARISA) and terminal restriction fragment length polymorphism (T-RFLP) of phylogenetic marker genes (Needham et al., 2013; Chow et al., 2014). These reports were able to show particular temporal dynamics among some members of the studied communities. Here we have assessed the role of biogeographical and limnological patterns in driving community composition, as well as global co-variation among the different components of microbial life. Overall, the results derived from this study support previous reports on the composition of polar freshwater microbial communities. They are also in agreement with environmental filtering representing a predominant factor driving community structure, with geographical distance effects appearing only at large scales. Also, the noticeable fraction of shared OTUs among sites seemingly indicates reduced dispersal limitations for microorganisms in these ecosystems (Logares et al., 2013). The availability of deep-sequencing phylogenetic marker genes data has allowed the study of community structure no only based on OTUs' relative abundance, but also on overall phylogenetic content. The instances when these two complementary approaches have disagreed have provided important information regarding the ecology of the ecosystems. For example, compositional shifts along the narrow limnological space sampled at the Antarctic sites were only detected based on OTUs' relative abundances, but not when taking into account their phylogenetic relationships. Since ecological coherence and gene conservation have been shown to be negatively correlated with phylogenetic distance (Philippot et al., 2010; Zaneveld et al., 2010), a plausible explanation is that small differences in limnological parameters shifted OTU-level community structure, yet the overall link between ecological function and phylogenetic structure remained stable.

## Author contributions

AA, DA, and DP conceived the study. DA undertook all wet-lab procedures. DA, CP analyzed the data and wrote the manuscript, with input from the other contributors.

### Conflict of interest statement

The authors declare that the research was conducted in the absence of any commercial or financial relationships that could be construed as a potential conflict of interest.
