# Supplementary material for: Composition and Interactions among Bacterial, Microeukaryotic, and T4-like Viral Assemblages in Lakes from Both Polar Zones
Source: Front Microbiol. 2016 Mar 18;7:337. doi: 10.3389/fmicb.2016.00337 (PMC4796948; doi:10.3389/fmicb.2016.00337)
Supplement: Supplementary file 1 [file Table1.DOCX]

| **Supplementary Table 1. Number of reads, OTUs (97%), singletons and Chao 1 estimate for every layer of every freshwater body.** |  |
| --- | --- |

| **Lake** | **Fraction** | **# Seqs** | **OTUs** | **Singletons** | **Chao1** |
| --- | --- | --- | --- | --- | --- |
| Domo | 16S | 6 130 | 195 | 89 | 314 |
|  | 18S | 1 725 | 113 | 57 | 186 |
|  | g23^f^ | na | na | na | na |
|  | g23^c^ | 1 436 | 51 | 20 | 99 |
| Refugio | 16S | 3 871 | 651 | 320 | 1 091 |
|  | 18S | 1 927 | 228 | 116 | 404 |
|  | g23^f^ | na | na | na | na |
|  | g23^c^ | na | na | na | na |
| Limnopolar | 16S | 3 777 | 264 | 134 | 499 |
|  | 18S | 2 007 | 160 | 73 | 240 |
|  | g23^f^ | 1 577 | 21 | 5 | 24 |
|  | g23^c^ | 2 023 | 16 | 6 | 31 |
| Cierva | 16S | 6 887 | 271 | 104 | 399 |
|  | 18S | 1 751 | 257 | 118 | 414 |
|  | g23^f^ | na | na | na | na |
|  | g23^c^ | 2 002 | 6 | 1 | 6 |
| Green | 16S | 4 995 | 487 | 192 | 695 |
|  | 18S | 3 100 | 205 | 100 | 332 |
|  | g23^f^ | 1 450 | 53 | 19 | 70 |
|  | g23^c^ | 2 032 | 35 | 12 | 42 |
| Biscoe | 16S | 6 213 | 301 | 119 | 454 |
|  | 18S | 1 294 | 87 | 41 | 133 |
|  | g23^f^ | 1 041 | 5 | 3 | 7 |
|  | g23^c^ | 1 293 | 15 | 3 | 17 |
| Pourquoi-Pas | 16S | 4 650 | 157 | 75 | 355 |
|  | 18S | 1 545 | 146 | 69 | 284 |
|  | g23^f^ | 1 680 | 16 | 5 | 18 |
|  | g23^c^ | 1 185 | 20 | 7 | 24 |
| Avian | 16S | 5 202 | 256 | 129 | 473 |
|  | 18S | 1 841 | 269 | 133 | 415 |
|  | g23^f^ | 1 861 | 41 | 13 | 51 |
|  | g23^c^ | 1 207 | 48 | 18 | 62 |
| Horseshoe | 16S | 6 980 | 126 | 46 | 191 |
|  | 18S | 1 572 | 121 | 54 | 186 |
|  | g23^f^ | 2 058 | 21 | 8 | 30 |
|  | g23^c^ | 2 484 | 12 | 4 | 14 |
| IR2 | 16S | 4 721 | 742 | 472 | 1 584 |
|  | 18S | 1 529 | 418 | 249 | 781 |
|  | g23^f^ | 2 098 | 10 | 0 | 10 |
|  | g23^c^ | 730 | 11 | 1 | 11 |
| Tunsijøen IR1 | 16S | 4 841 | 319 | 143 | 531 |
|  | 18S | 1 530 | 241 | 143 | 495 |
|  | g23^f^ | 633 | 6 | 0 | 6 |
|  | g23^c^ | 760 | 11 | 4 | 13 |
| Tenndamen SvL2 | 16S | 4 744 | 722 | 362 | 1 206 |
|  | 18S | 1 978 | 87 | 50 | 189 |
|  | g23^f^ | 1 537 | 13 | 2 | 14 |
|  | g23^c^ | 1 214 | 13 | 2 | 14 |
| Nordammen SvL1 | 16S | 5 920 | 864 | 462 | 1 466 |
|  | 18S | 1 674 | 226 | 116 | 393 |
|  | g23^f^ | 2 017 | 20 | 8 | 48 |
|  | g23^c^ | 1 353 | 159 | 73 | 241 |
| **Average** | 16S | 5 302 | 412 | 204 | 712 |
|  | 18S | 1 806 | 197 | 101 | 342 |
|  | g23^f^ | 1 595 | 21 | 6 | 28 |
|  | g23^c^ | 1 477 | 33 | 13 | 48 |

**^f^**Free virus fraction. **^c^**Cellular fraction.
