# Supplementary material for: Composition and Interactions among Bacterial, Microeukaryotic, and T4-like Viral Assemblages in Lakes from Both Polar Zones
Source: Front Microbiol. 2016 Mar 18;7:337. doi: 10.3389/fmicb.2016.00337 (PMC4796948; doi:10.3389/fmicb.2016.00337)
Supplement: Supplementary file 2 [file Table2.DOCX]

| **Supplementary Table 2.** Limnological **values recorded for the Antarctic lakes.** | | | | |
| --- | --- | --- | --- | --- |
| Lake | **Temp (ºC)** | **Conductivity (µS.cm^-1^)** | **pH** | **Chl_a_ (µg.L^-1^)** |
| **Domo** | 3.61 | 35 | 6.86 | 3.5 |
| **Refugio** | 3.98 | 426 | 7.75 | 120.3 |
| **Limnopolar** | 2.56 | 49 | 7.14 | 4.3 |
| **Cierva** | 1.46 | 6 | 7.51 | 3.8 |
| **Biscoe** | 2.91 | 47 | 6.67 | 4.5 |
| **Pourquois-Pas** | 4.58 | 185 | 5.97 | 4.7 |
| **Horseshoe** | 1.8 | 69 | 5.85 | 3.1 |
| **Avian** | 4.77 | 150 | 6.74 | 5.9 |
