# Supplementary material for: Composition and Interactions among Bacterial, Microeukaryotic, and T4-like Viral Assemblages in Lakes from Both Polar Zones
Source: Front Microbiol. 2016 Mar 18;7:337. doi: 10.3389/fmicb.2016.00337 (PMC4796948; doi:10.3389/fmicb.2016.00337)
Supplement: Supplementary file 3 [file Image1.PDF]

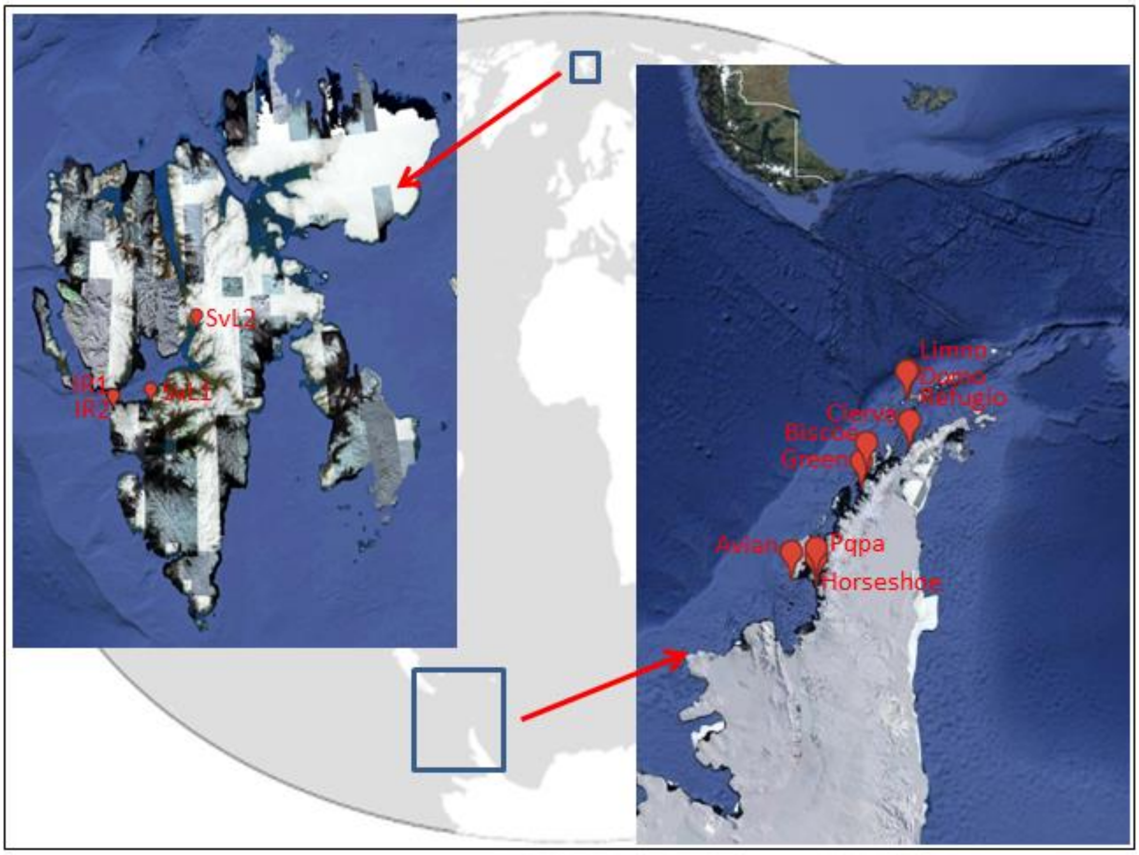

Supplementary Figure 1. Studied environments. The composite map images represent the location of the studied environments along the Antarctic Peninsula and Spitsbergen Island. Red symbols indicate sampling sites. Maps obtained from Google; Inav/Geosistemas SRL (Antarctica)/ NASA, TerraMetrics (Svalvard).
